# Combination therapy with gefitinib and doxorubicin inhibits tumor growth in transgenic mice with adrenal neuroblastoma

**DOI:** 10.1002/cam4.76

**Published:** 2013-04-02

**Authors:** Kumi Kawano, Yoshiyuki Hattori, Hiroshi Iwakura, Takashi Akamizu, Yoshie Maitani

**Affiliations:** 1 Institute of Medicinal Chemistry, Hoshi UniversityEbara 2-4-41, Shinagawa-ku, Tokyo, 142-8501, Japan; 2 Medical Innovation Center, Kyoto University Graduate School of MedicineYoshida-Konoe-Cho, Sakyo-ku, Kyoto, 606-8501, Japan; 3 The First Department of Medicine, Wakayama Medical University811-1 Kimi-idera, Wakayama, 641-8509, Japan

**Keywords:** Adrenal neuroblastoma, doxorubicin, gefitinib, liposome, transgenic mouse

## Abstract

Highly relevant mouse models of human neuroblastoma (NB) are needed to evaluate new therapeutic strategies against NB. In this study, we characterized transgenic mice with bilateral adrenal tumors. On the basis of information from the tumoral gene expression profiles, we examined the antitumor effects of unencapsulated and liposomal doxorubicin (DXR), alone and in combination with gefitinib, on adrenal NB. We showed that intravenous injection of unencapsulated or liposomal DXR alone inhibited tumor growth in a dose-dependent manner, as assessed by magnetic resonance imaging (MRI). However, liposomal DXR did not exhibit greater antitumor effect than unencapsulated DXR. Immunohistochemical analysis revealed that the adrenal tumor vasculature with abundant pericyte coverage was a less leaky structure for liposomes. Combination therapy with unencapsulated or liposomal DXR plus gefitinib strongly suppressed tumor growth and delayed tumor regrowth than treatment with unencapsulated or liposomal DXR alone, even at a lower dose of DXR. Dynamic contrast-enhanced MRI analysis revealed that gefitinib treatment increased blood flow in the tumor, indicating that gefitinib treatment changes the tumor vascular environment in a manner that may increase the antitumor effect of DXR. In conclusion, the combination of gefitinib and DXR induces growth inhibition of adrenal NBs in transgenic mice. These findings will provide helpful insights into new treatments for NB.

## Introduction

Neuroblastoma (NB) is the most common extracranial solid tumor of children, and the neoplasm is most frequently diagnosed during infancy [1]. Despite improvements in chemotherapy, the prognosis is poor for the majority of children over 1 year of age with advanced-stage NB [2]. Although in vitro preclinical research has identified several agents with promising therapeutic potential for the treatment of this malignancy, the in vivo efficacy of these candidates is limited by insufficient drug delivery to the tumor and high toxicity [3–5]. Therefore, liposome-based drug delivery systems are a promising strategy for increasing the efficacy of cytotoxic drugs against NB, as liposomes carry large drug cargos and passively accumulate in tumor tissues due to the leaky structure of tumor vasculature; this accumulation is known as the enhanced permeability and retention (EPR) effect [6]. Indeed, liposomal anti-cancer drugs have shown antitumor efficacy against NB in human tumor xenografts [7] and an orthotopic animal model [8].

Model animals that closely and reliably mimic the human tumor microenvironment are needed to evaluate the antitumor activities and develop effective therapeutic regimens involving liposomal anti-cancer drugs and drugs whose effects, such as antiangiogenesis, require interaction with the blood vessels, as the usefulness of such drugs depends on the tumor microenvironment [9]. Mouse models with subcutaneously or orthotopically implanted tumors have often been used to evaluate the antitumor effects of anti-cancer drugs; however, implanted tumors differ in character from spontaneous tumors because the implanted tumors do not grow under physiological conditions [10]. The tumor microenvironment is relevant to the clinical prognosis, metastatic potential, and treatment-related outcomes of tumors. Recent technological advances in the creation of transgenic mice have allowed the development of many kinds of cancer models. Transgenic mice are valuable models for testing cytotoxic chemotherapy regimens. Recently, Teitz et al. reported that TH-NMYC transgenic mice, in which N-myc expression is driven by the rat tyrosine hydroxylase promoter, are a useful preclinical model of NB; however, these transgenic mice developed tumors in the paraspinal ganglia rather than in the adrenal glands [11], whereas approximately 40% of patient tumors originate in the adrenal medulla [1].

We have previously generated transgenic mice expressing the SV40 T-antigen that develop tumors only in the adrenal glands [12]. The histological characteristics and DNA microarray gene expression profiles of the adrenal tumors of these transgenic mice are similar to those of human adrenal NB [12, 13]. Furthermore, DNA microarray analysis showed overexpression of DNA topoisomerase II (Topo IIα) and epidermal growth factor receptor (EGFR) in the adrenal NBs of the transgenic mice [13]. On the basis of this information from the gene profiles, we hypothesized that doxorubicin (DXR) and gefitinib, which inhibit Topo IIα and EGFR, respectively, might be effective treatments for NB.

In this study, we characterized the adrenal tumors of transgenic mice and evaluated the antitumor activities of DXR and liposomal DXR alone and in combination with gefitinib in a transgenic mouse model of adrenal NB in order to advance the development of novel chemo-therapeutic regimens.

## Materials and Methods

### Materials

Hydrogenated soybean phosphatidylcholine (HSPC) and methoxy-poly(ethylene glycol)-distearoylphosphatidylethanolamine (mPEG-DSPE; mean molecular weight of PEG: 2000) were purchased from NOF (Tokyo, Japan). Cholesterol and DXR hydrochloride were obtained from Wako Pure Chemical Industries, Ltd. (Osaka, Japan).

### Animals

Transgenic mice on a C57BL/6 background that exhibit spontaneous bilateral adrenal tumors were previously described [12]. The transgenic mice carried tetracycline-inducible simian virus 40 T-antigen, a fusion gene comprising tetracycline-responsive elements, the cytomegalovirus promoter, and simian virus 40 T-antigen. Heterozygous transgenic mice were used for the experiments. All experimental procedures were approved by the Committee on Animal Research of Hoshi University.

### Quantitative RT-PCR analysis

Total RNA was isolated using the RNeasy Midi Kit (Qiagen, Hilden, Germany) from adrenal tumors of transgenic mice at the ages of 5, 9, 13, 15, and 17 weeks and from normal adrenal glands from nontransgenic littermates at the ages of 5 and 13 weeks. Mouse topoisomerase (Topo) IIα cDNA was amplified using the primers Topo IIα-FW, 5′-GAACAGTCAAAGCCATCC-3′, and Topo IIα-RW, 5′-GAATCTGATTTGGTTCCC-3′. Mouse EGFR cDNA was amplified using the primers EGFR-FW, 5′-TCTTCAAGGATGTGAAGTGTG-3′, and EGFR-RW, 5′-GTACGCTTTCGAACAATGT-3′, as previously reported [14]. Mouse β-actin cDNA was amplified using the primers β-actin-FW, 5′-TGTGATGGTGGGAATGGGTCAG-3′, and β-actin-RW, 5′-TTTGATGTCACGCACGATTTCC-3′, as previously reported [15]. Quantitative reverse transcription polymerase chain reaction (RT-PCR) was performed with the iCycler MyiQ detection system (Bio-Rad Laboratories, Hercules, CA) and the SYBR Green I assay (iQ™ SYBER Green Supermix, Bio-Rad Laboratories), as previously described [13]. Samples were run in triplicate, and the mRNA expression levels of Topo IIα and EGFR were normalized to the amount of β-actin mRNA in the same sample. A difference of one cycle was considered to represent a twofold change in gene expression.

### Liposome preparation

Liposomes loaded with DXR (liposomal DXR) were prepared as follows. A lipid film composed of HSPC/cholesterol (55/45, molar ratio) was hydrated with citrate buffer (300 mmol/L, pH 4.0) at 60°C, and the particle size was adjusted by sonication or by repeated extrusion through a polycarbonate membrane (Nuclepore™, Whatman®, GE Healthcare UK Ltd., Buckinghamshire, U.K.). Surface modification with mPEG-DSPE (at 2.5 mol% of total lipids) was performed by the postinsertion technique [16]. The liposomes were then actively loaded with DXR by a pH gradient method [17]. The external pH was adjusted to 7.4, and the liposomes were incubated with DXR (1/5 drug/lipid [w/w]) at 60°C for 25 min. The DXR loading efficiency was greater than 95%. The mean particle size, as determined by the dynamic light scattering method (ELS-Z2, Otsuka Electronics Co., Ltd., Osaka, Japan), was approximately 120–130 nm.

### In vivo therapeutic experiments and DXR biodistribution

Treatment was started at 13 weeks of age; this is the age at which the adrenal tumors begin to grow [13]. Mice were treated at weekly intervals with three intravenous injections of unencapsulated or liposomal DXR at a DXR dose of 3, 5, or 8 mg/kg per injection. Control mice were injected intravenously with saline. For combination therapy with unencapsulated or liposomal DXR plus gefitinib, gefitinib was administered orally at 100 mg/kg per day for five consecutive days, and unencapsulated or liposomal DXR was then administered intravenously at 5 mg/kg on the fifth day of gefitinib treatment.

Adrenal tumor sizes were determined by magnetic resonance imaging (MRI) as previously described [18]. Briefly, anesthetized mice (1.5% isoflurane) were inserted into a 9.4-T vertical-type MRI scanner (Varian MRI System, Varian, Palo Alto, CA). Coronal T_2_-weighted images were obtained using a multislice fast-spin echo sequence (repetition time/effective echo time: 2500 msec/48 msec) with a field of view of 50 × 30 mm, a matrix of 256 × 256 pixels, and a slice thickness of 1 mm. The adrenal glands were selected as the regions of interest (ROIs) using ImageJ software (NIH, Bethesda, MD), and the volume of each adrenal gland was calculated by summing the ROIs in consecutive slices. The adrenal tumor size was measured from MR images from 13 to 22 weeks of age; this method has previously been shown to correlate well with the wet tumor weight (*r* = 0.996) and detect tumors as small as ∼1.5 mm in diameter [18].

The biodistributions of unencapsulated and liposomal DXR (5 mg/kg) were evaluated in 14-week-old mice. The schedule used for the combination therapy was the same as that used for the treatment described previously. At the indicated time points, tumors and tissues were excised and homogenized in 0.1 mol/L NH_4_Cl/NH_3_ buffer (pH 9.0). DXR was extracted with a mixture of chloroform and methanol (2:1, v/v) and quantified by HPLC [19].

### Histological examination and immunohistochemistry

Transgenic mice were treated with either unencapsulated or liposomal DXR (5 mg/kg) at 14 weeks of age. One week after the treatment, the tumors were excised and immediately frozen. The tumor sections (20 μm thick) were stained with hematoxylin and pure eosin (H&E staining) (Muto Pure Chemicals Co., Ltd., Tokyo, Japan).

To detect mouse endothelial cells, the adrenal tumor sections from a transgenic mouse (17 weeks old) were incubated with rat anti-mouse CD31 (PECAM-1) monoclonal antibody (diluted 1:50; Clone MEC 13.3, BD Pharmingen, San Diego, CA). Tumor sections of human NBs (US Biomax, Inc., Rockville, MD) were incubated with mouse anti-human CD34 monoclonal antibody (diluted 1:200; Clone QBEnd/10, Thermo Fisher Scientific, Inc., Fremont, CA). These sections were subsequently incubated for another 1 h with Alexa Fluor 488-conjugated secondary antibodies (Invitrogen, Carlsbad, CA). To detect mouse and human pericytes, the sections were incubated with Cy3-conjugated rabbit anti-smooth muscle α-actin (α-SMA) antibody (diluted 1:200; C6198, Sigma-Aldrich, MO) for 1 h.

For observing blood vessel function, the transgenic mice treated with or without gefitinib were intravenously injected with the perfusion marker Hoechst 33342 (15 mg/kg; Invitrogen) 3 min before killing them. Excised tumors were immediately frozen and sectioned at a thickness of 20 μm. The specimens were examined microscopically using an ECLIPSE TS100 microscope (Nikon Corp., Tokyo, Japan).

### Dynamic contrast-enhanced MRI

To observe the hemodynamic characteristics of the adrenal tumors, dynamic contrast-enhanced (DCE)-MRI was performed on mice treated with or without gefitinib at 100 mg/kg per day for five consecutive days. DCE-MRI acquisition was performed repeatedly to acquire axial-slice spoiled gradient-recalled echo images at a 1-sec temporal resolution over 6 min, with repetition time = 7.8125 msec, echo time = 2.06 msec, matrix resolution = 64 × 64, field of view = 30 × 30 mm^2^, slice thickness = 3 mm, flip angle = 30, number of slices = 1, and two signal averages, as described previously [20]. Gd-DTPA (Magnevist®, Bayer-Schering Pharma AG, Berlin, Germany) was used as an MRI contrast agent and was administered as a bolus at 0.1 mmol Gd/kg in heparinized saline (total volume, ∼0.4 mL). Serial MR images were acquired before, during, and after intravenous injection of Gd-DTPA. The tumor concentration of Gd at each imaging time point was estimated from the change in signal intensity after contrast injection [20].

### Statistical analysis

Survival was plotted as Kaplan–Meier survival curves, and the statistical significance was determined by the Log-rank test. The statistical significance of tumor size was determined by the Student's *t*-test for two groups or by the Kruskal–Wallis test followed by Dunn's post-test for more than two groups. Differences for which *P* was <0.05 were considered statistically significant. All statistical computations were performed using GraphPad Prism (GraphPad Software, Inc., CA).

## Results

### Expression levels of Topo IIα and EGFR mRNA in adrenal NB

**Figure 1:**
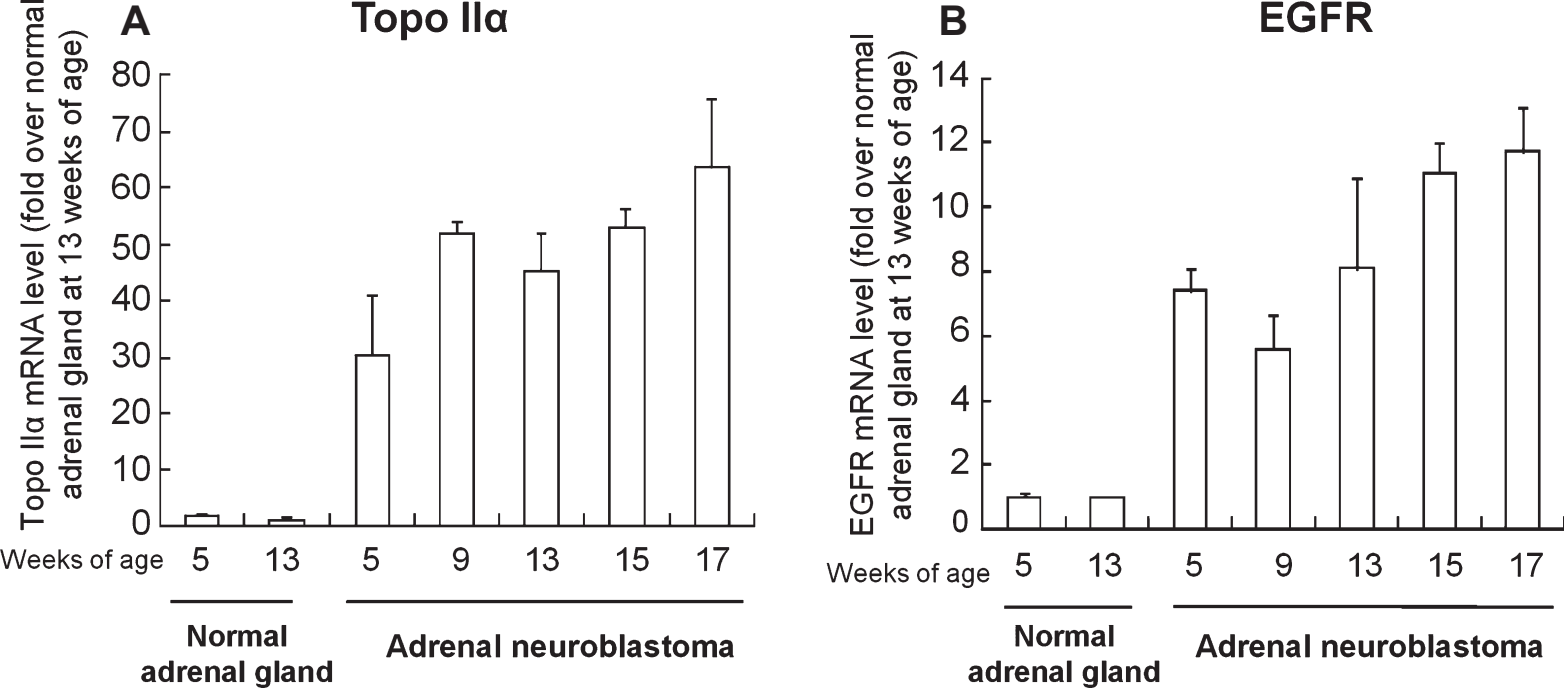
Quantitative RT-PCR analysis of the mRNA expression levels of Topo IIα (A) and epidermal growth factor receptor (EGFR) (B) in the adrenal tumors from the transgenic mice. mRNA was purified from the adrenal tumors of transgenic mice aged 5–17 weeks and the adrenal glands of nontransgenic littermates aged 5 and 13 weeks. Each result represents the mean ± standard deviation (SD) (*n* = 3).

Genome-wide gene expression studies can provide insight into the genes and molecular pathways that govern tumor pathogenesis and can also highlight therapeutic targets. We previously found by microarray analysis that Topo IIα and EGFR were overexpressed at the mRNA level in adrenal NBs of transgenic mice [13]. Here, we quantitatively measured the expression levels of these genes by quantitative RT-PCR analysis. Increased mRNA expression levels of Topo IIα and EGFR were observed in the adrenal glands of our transgenic mice beginning at 5 weeks of age, and the expression levels at 9–17 weeks of age were about 50- to 70-fold and 6- to 12-fold higher, respectively, than those of normal adrenal glands (Fig. 1A and B). This finding suggested that these adrenal tumors might be highly sensitive to DXR and gefitinib.

### Antitumor effects of unencapsulated and liposomal DXR

**Figure 2:**
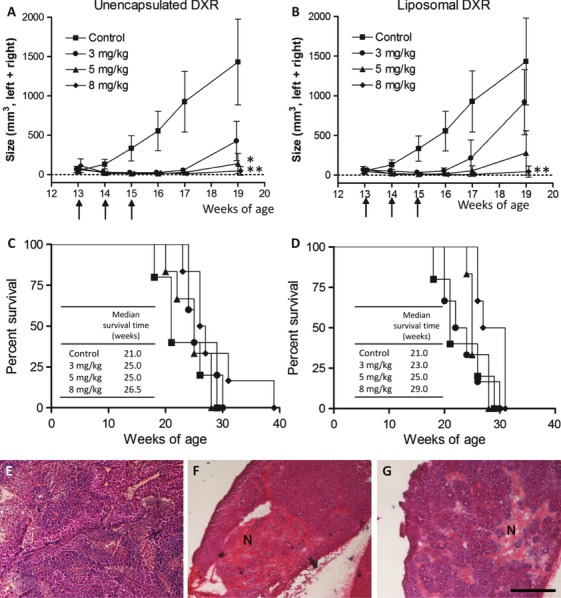
Antitumor effects of unencapsulated and liposomal DXR (A–D) and histological analysis after the treatment (E–G). Adrenal tumor size (A and B) and survival time (C and D) after treatment with unencapsulated doxorubicin (DXR) (A and C) or liposomal DXR (B and D). The arrows indicate the times of drug treatment. The tumor size represents the sum of the volumes of the left and right adrenal glands calculated from MR images. Each value represents the mean ± SD (*n* = 5–6). *P < 0.05, **P < 0.01 versus control mice at 19 weeks of age. (E–G) Histological analysis of the adrenal tumors 1 week after injection of saline (E), unencapsulated DXR (F), or liposomal DXR (G) at 5 mg/kg into 14-week-old transgenic mice. The sections were stained with H&E. N, necrotic area. Scale bar = 500 μm.

Liposomal DXR has been reported to be effective for the treatment of solid tumors [21]. Therefore, we evaluated and compared the antitumor activities of unencapsulated and liposomal DXR against the adrenal tumors of transgenic mice. Unencapsulated or liposomal DXR was injected intravenously into transgenic mice at doses of 3–8 mg/kg once weekly for three successive weeks (Fig. 2A and B). All mice treated with unencapsulated or liposomal DXR at 8 mg/kg showed significant tumor suppression at 19 weeks relative to the saline-treated control mice (*P* < 0.01). The tumor-suppressive activity of DXR in these mice was prolonged and dose-dependent. Surprisingly, tumor growth did not differ significantly between mice treated with unencapsulated and liposomal DXR. Furthermore, at equivalent doses (8 mg/kg), both formulations slightly prolonged the median survival times of treated mice (26.5 weeks for unencapsulated DXR and 29 weeks for liposomal DXR) relative to those of control mice (21 weeks; Fig. 2C and D).

Next, we histologically examined adrenal tumors 1 week after a single intravenous injection of unencapsulated or liposomal DXR (Fig. 2E–G). The areas of necrosis, which were not stained by hematoxylin, were larger in the sections from the mice injected with unencapsulated DXR (22.1%) than in those from the mice injected with liposomal DXR (15.5%). The results indicated that un-encapsulated DXR showed a slightly higher antitumor effect than that of liposomal DXR.

### Biodistribution of liposomal DXR

**Figure 3:**
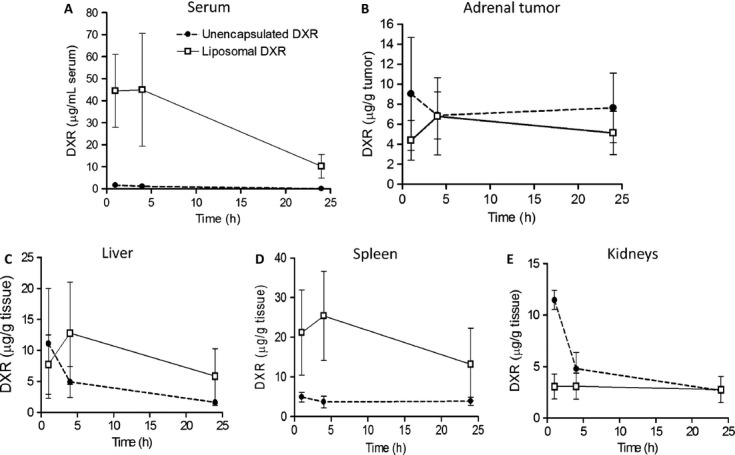
DXR levels in the serum (A), adrenal tumors (B), liver (C), spleen (D), and kidneys (E) after intravenous injection of unencapsulated DXR (•) or liposomal DXR (□) at 5 mg/kg into 14-week-old transgenic mice. Each value represents the mean ± SD (*n* = 3, serum and tissues; *n* = 6, tumor).

To investigate why the antitumor activity of liposomal DXR was no greater than that of unencapsulated DXR, we measured the DXR levels in the adrenal tumors and normal tissues after intravenous injection of the transgenic mice with unencapsulated or liposomal DXR at a dose of 5 mg/kg (Fig. 3). Liposomal DXR exhibited prolonged blood circulation: 24 h postinjection, 10.2 ± 5.4% of the dose of DXR remained in the blood of mice treated with liposomal DXR, whereas circulating DXR was below the detection limit in mice treated with unencapsulated DXR (Fig. 3A). Liposomal DXR accumulated in the liver and spleen to a greater degree than did unencapsulated DXR (Fig. 3C and D). Prolonged circulation in the blood and high hepatic and splenic accumulation of the encapsulated drug are characteristic of PEGylated liposomes. The accumulation of liposomal DXR in the tumor was similar to that of unencapsulated DXR (Fig. 3B). This finding was in contrast to early reports that liposomal DXR accumulated to a greater extent than did unencapsulated DXR in subcutaneously inoculated tumors [21, 22]. To clarify this discrepancy, we examined the vascular structure of adrenal tumors.

### Vascular structure of adrenal NBs

**Figure 4:**
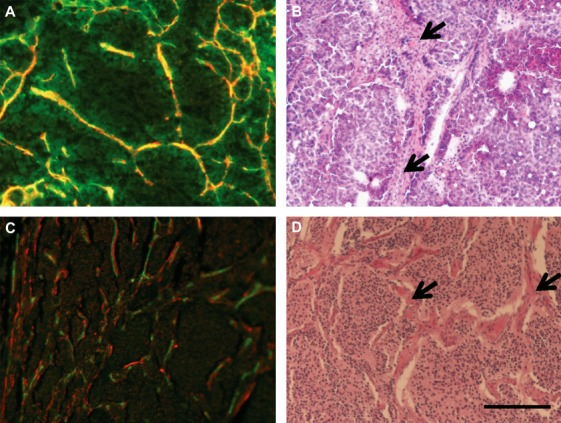
Immunostaining for endothelial cells and pericytes (A and C) and H&E staining (B and D) of mouse adrenal tumors (A and B) and human neuroblastoma (C and D). In (A) and (C), the green signals indicate endothelial cells; and the red signals, α-SMA-positive pericytes. The arrows in (B) and (D) indicate stromal cells. Scale bar = 200 μm.

Tumor accumulation of nanocarriers by the EPR effect is limited by the tumor microenvironment, including vascularity, permeability, or blood flow [23]. We characterized the vascular structure of these adrenal tumors by immunohistochemical analysis and compared it with that of NBs from human patients. Examination of tumor sections from the transgenic mice showed that most of the CD31-positive endothelial cells were well covered with α-SMA-positive pericytes (Fig. 4A). Coverage of vasculature by pericytes stabilizes vascular structure [24]. Therefore, the vasculature of the adrenal tumors was relatively stable and nonleaky. Similarly, the representative tumor sections of human NBs also showed CD34-positive endothelial cells covered by abundant α-SMA-positive cells (Fig. 4C). H&E staining frequently showed stromal cells in the tumor sections of both human and mouse adrenal NBs (Fig. 4B and D). These findings indicated that the histological appearance of the NBs of our transgenic mice had a similarity to that of human NBs in terms of the extensive pericyte coverage of the vasculature, which may be related to an inability of liposomal DXR to accumulate and distribute into the tumor tissue. In addition, unencapsulated DXR might have some affinity with adrenal tumors, resulting in sustained retention in adrenal tumors.

### Neuroblastoma tumor growth inhibition by combination therapy with DXR and gefitinib

**Figure 5:**
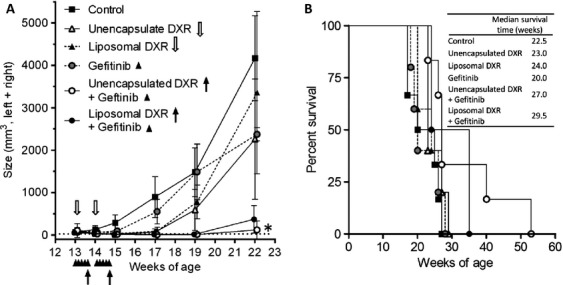
Adrenal tumor size (A) and survival time (B) after treatment with unencapsulated or liposomal DXR in combination with gefitinib for 2 weeks. Transgenic mice were orally administered gefitinib at a dose of 100 mg/kg per day for five consecutive days with or without intravenous injection of unencapsulated or liposomal DXR at a dose of 5 mg/kg on the fifth day of gefitinib treatment. Arrows and arrow heads indicate the days of drug treatment. The tumor size represents the sum of the left and right adrenal gland volumes calculated from MR images. The tumor size values are given as the mean ± SD (*n* = 4–6). *P < 0.05 versus control at 22 weeks of age.

Quantitative RT-PCR confirmed that EGFR mRNA was overexpressed in the adrenal NBs of our transgenic mice (Fig. 1B). To investigate whether the EGFR inhibitor gefitinib could increase the antitumor effect of DXR against the adrenal tumors of transgenic mice, we evaluated the antitumor activity of gefitinib alone and in combination with unencapsulated or liposomal DXR. Although the treatment with gefitinib alone did not affect tumor growth, marked tumor suppression was observed in all the mice treated with unencapsulated or liposomal DXR plus gefitinib (Fig. 5A). Unencapsulated DXR plus gefitinib was especially effective and suppressed tumor growth at 22 weeks relative to saline treatment (*P* < 0.05) to a degree similar to that produced by three injections of unencapsulated DXR alone at 8 mg/kg (Fig. 2A). The median survival times were prolonged in mice treated with unencapsulated DXR plus gefitinib (27 weeks) or liposomal DXR plus gefitinib (29.5 weeks) relative to mice treated with either DXR formulation alone (23 and 24 weeks, respectively; Fig. 5B).

**Figure 6:**
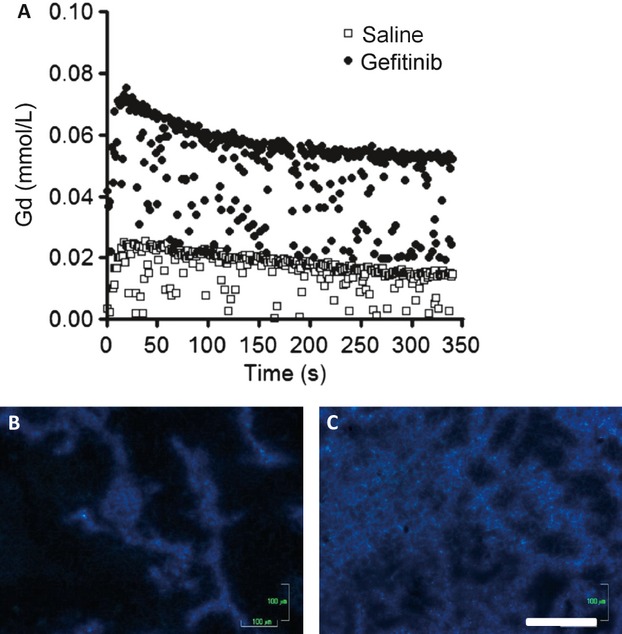
Tumor vascular change after the oral gefitinib treatment (100 mg/[kg·day] for five consecutive days), as determined by DCE-MRI (A) and Hoechst 33342 staining (B and C). (A) Tumor Gd concentration was assessed by DCE-MRI using Gd-DTPA. Each data point represents the dot plots of an individual mouse. (B and C) Tumor vessels stained by intravenous injection of Hoechst 33342 as a perfusion marker in the saline- (B) and gefitinib-treated mice (C). Scale bar, 200 μm.

Finally, to clarify the mechanism by which gefitinib increased the antitumor effect of DXR, we used DCE-MRI to observe the vascular function in the tumors after oral administration of gefitinib. Gd-DTPA accumulated progressively in the tumors of mice treated with gefitinib (Fig. 6A). From the DCE-MRI data, the hemodynamic parameters *K*^trans^ and *V*_p_ could not be obtained because of the lack of a suitable artery in the tumor field of view. For further evaluation, the tumor blood vessel was stained with an intravenous injection of Hoechst 33342, a DNA-binding dye that stains the nuclei of endothelial cells and cells adjacent to tumor blood vessels perfused at the time of injection, hence delineating the functional tumor vasculature. The tumor treated with gefitinib showed a larger area stained with Hoechst 33342 than did the control (Fig. 6B and C). These findings indicate that gefitinib temporarily changed the tumor microenvironment, such as by increasing blood flow and/or perfusion in the tumor. These results might explain the enhanced antitumor activity of the combination of gefitinib and DXR against NB in transgenic mice.

## Discussion

A transgenic mouse model of NB was used to evaluate the antitumor effects of DXR and gefitinib. Because liposomal DXR (Doxil, Janssen Products, LP, Horsham, PA) has been used clinically to deliver DXR into solid tumors via the EPR effect, we evaluated the antitumor activities of unencapsulated and liposomal DXR against the tumors in the transgenic mice. Injection of liposomal DXR at a dose of 8 mg/kg inhibited the growth of adrenal tumors similarly to injection of unencapsulated DXR and enhanced mean survival time (29 weeks) only slightly over that of mice injected with unencapsulated DXR (26.5 weeks; Fig. 2). This finding was consistent with those of several previous reports. For example, a comparison between Doxil and free DXR demonstrated that the former produced significantly less severe adverse effects and at least comparable survival times [25], and Doxil and free DXR at 5 mg/kg did not improve the survival of mice with orthotopic NB xenografts in the adrenal gland [7].

The permeability of the tumor vasculature to liposomes is well known to depend on the pericyte coverage of the vasculature [9]. In general, most tumor exhibit low pericyte coverage of vessels [24]. However, in the adrenal tumors of transgenic mice as well as human adrenal NB, most of the endothelial cells were covered with pericytes (Fig. 4). The characteristics of the vasculature of adrenal NBs probably prevent liposomal DXR from accumulating in the tumors by the EPR effect; therefore, unencapsulated and liposomal DXR resulted in similar degrees of tumor growth inhibition (Fig. 2). Another reason for the limited antitumor effect of liposomal DXR might be the slow release of drug from the liposome in the tumor tissue. Because drugs released from a liposome reveal activity, liposomal DXR resulted in less tumor suppression even though the accumulated amount of DXR was almost similar to that of unencapsulated DXR. As reported previously, the lifespan of mouse models of orthotopic implanted NB is significantly increased by modification in vascular targeting ligand with liposomal DXR, but not by unmodified liposomal DXR [7].

Tyrosine kinase receptors are important for the survival, growth, and differentiation of many normal and malignant cells. EGFR expression has been observed in NB cell lines and in primary tumors [26], and inhibition of EGFR phosphorylation by gefitinib-induced apoptosis in NB cell lines [27, 28], suggesting that gefitinib might be a potent inhibitor of NB cell proliferation. As EGFR mRNA was overexpressed in the adrenal NBs of transgenic mice (Fig. 1B), we examined the therapeutic effect of gefitinib in the transgenic mice (Fig. 5). However, oral treatment with gefitinib alone could not suppress tumor growth. This result agrees with that of a previous report that gefitinib used alone has limited activity in vivo against NB xenografts [29].

In clinical use, the addition of gefitinib to irinotecan [30] or cyclophosphamide plus topotecan [31] improved the therapeutic response in children with high-risk NB. In human NBs, Topo IIα overexpression was reported previously [32]. Therefore, we evaluated the antitumor activity of the combination therapy with unencapsulated or liposomal DXR plus gefitinib and found markedly inhibited tumor growth at 22 weeks of age in all mice treated with either formulation of DXR plus gefitinib compared with the mice treated with unencapsulated or liposomal DXR alone (Fig. 5A). A combination of DXR and gefitinib has been reported to increase the growth inhibition of A549 xenografts 10-fold in an EGFR expression-independent manner [33]. We confirmed that oral treatment with gefitinib did not affect the blood concentration of DXR 24 h after intravenous injection of unencapsulated or liposomal DXR (Fig. S1). Moreover, no increased expression of ABCG2 (breast cancer resistance protein, BCRP) was observed in the NBs by DNA microarray analysis (data not shown). These findings suggest that the enhancement of the therapeutic response by gefitinib treatment is unlikely to be related to inhibition of the ABC transporter.

Gefitinib treatment has been reported to improve tumor vascular function and increase the rate of blood flow in tumors [33–35]. Gefitinib temporarily increased the blood flow in the adrenal tumors, as assessed by DCE-MRI and Hoechst 33342 staining (Fig. 6), suggesting that the effect of gefitinib on the tumor microvascular environment may increase the delivery of unencapsulated or liposomal DXR to the tumors. However, we could not rule out the possibility that gefitinib potentiates the cytotoxic action of DXR by inhibiting EGFR signaling in adrenal tumor cells [36]. Our results suggest that combination therapy with DXR and gefitinib is a promising novel strategy against NB.

In conclusion, we found that the combination of gefitinib and DXR resulted in enhanced antitumor activity against the adrenal NBs of transgenic mice. The results reported herein suggest that this treatment regime warrants further preclinical studies for use against adrenal NB. Further studies will be needed to investigate how the therapeutic efficacy of gefitinib can be further enhanced and determine the most appropriate combination of cytotoxic agents for the treatment of NB. Nevertheless, the level of gene expression may be considered as an indicator of the efficacy of combination therapy.

## Supporting Information

**Figure S1.** Serum DXR levels 24 h after injection of DXR and gefitinib. Gefitinib was administered orally at 100 mg/kg per day for five consecutive days, and unencapsulated or liposomal DXR was administered intravenously at 5 mg/kg on the fifth day of gefitinib treatment. Each value represents the mean ± SD (*n* = 3).
