# Supplementary figures and images for: Combination therapy with gefitinib and doxorubicin inhibits tumor growth in transgenic mice with adrenal neuroblastoma
Source: Cancer Med. 2013 Apr 2;2(3):286–95. doi: 10.1002/cam4.76 (PMC3699840; doi:10.1002/cam4.76)

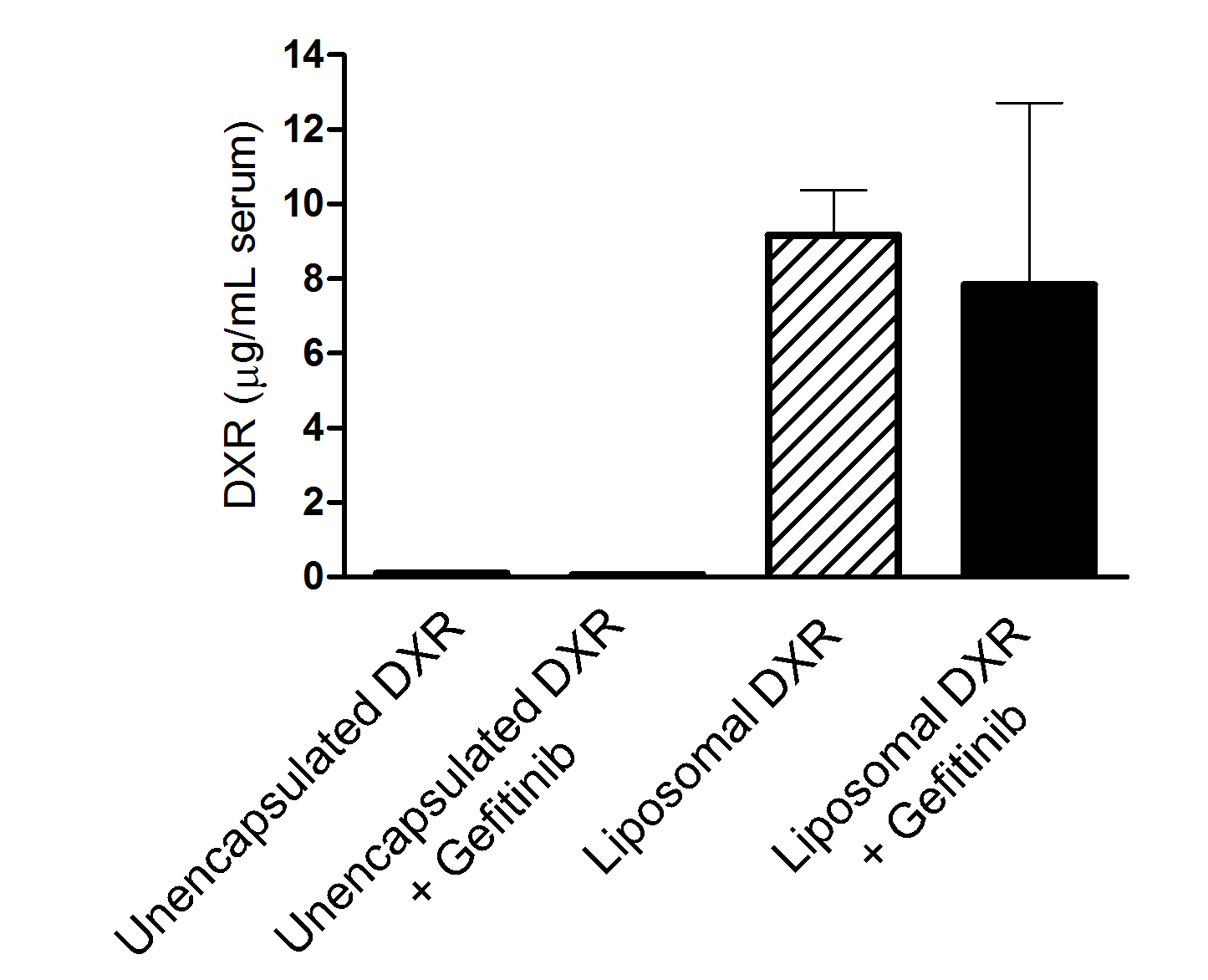

Supplement: Supplementary file 1 [file cam40002-0286-SD1.tif]
